# Serious hepatotoxicity following use of isoniazid preventive therapy in HIV patients in Eritrea

**DOI:** 10.1002/prp2.423

**Published:** 2018-07-31

**Authors:** Mulugeta Russom, Merhawi Debesai, Mehari Zeregabr, Araia Berhane, Theodros Tekeste, Teklezghi Teklesenbet

**Affiliations:** ^1^ Eritrean Pharmacovigilance Centre Asmara Eritrea; ^2^ Communicable Disease Control Division Asmara Eritrea; ^3^ Disgsa Community Hospital Segeneity Eritrea; ^4^ Keren Zonal Referral Hospital Keren Eritrea; ^5^ Present address: WHO Eritrea

**Keywords:** Eritrea, hepatotoxicity, isoniazid preventive therapy, patients on HAART

## Abstract

WHO information note indicates that isoniazid preventive therapy (IPT) is generally safe with little risk of hepatotoxicity. However, when the policy of IPT for HIV patients was introduced in Eritrea, frequent IPT‐associated hepatotoxicity and fatality have been reported to the Pharmacovigilance Centre. The aim of the study is to assess the causal association of IPT and hepatotoxicity and identify possible risk factors in patients on Highly Active Anti‐retroviral Therapy (HAART). This is a case series assessment of spontaneously reported cases to the Eritrean Pharmacovigilance Centre. Data extracted from VigiFlow (reported between 2014 and 2016) were exported to excel spread sheet for descriptive and qualitative analysis. Naranjo probability scale and Austin Bradford‐Hill criteria were used to assess causality. The P‐Method was used to assess preventability. A total of 31 of cases of hepatotoxicity related to IPT were retrieved. Majority (80.6%) of the cases were marked as “serious” due to life‐threatening situation (n = 15), hospitalization (n = 6), and death (n = 4). Baseline liver function test was normal in 61.3% and hepatitis B and C infections were ruled out in 77.4%. IPT was discontinued in 26 cases and reaction abated in 22 of them. Causality assessment using Austin Bradford‐Hill criteria found that the association was strong, consistent and specific with a plausible temporal relationship and biological mechanism. IPT‐associated hepatotoxicity that led to treatment interruption and death was observed in patients on HAART in Eritrea. Hence, close laboratory monitoring of patients is recommended to minimize the risk.

**Abbreviations**
ADR: adverse drug reaction
ALT: alanine aminotransferase
AST: aspartate aminotransferase
CYP1A2: cytochrome P1A2
HAART: highly active anti‐retroviral therapy
HIV: human immunodeficiency virus
ICH: international conference for harmonization
INH: isonicotinyl hydrazide
IPT: isoniazid preventive therapy
IU: international unit
LFT: liver function test
PLWH: people living with HIV
PRR: proportional reporting ratio
TB: tuberculosis
ULN: Upper Limit Normal
US‐FDA: United States food and drug administration
WHO: World health organization

## 1 INTRODUCTION

Tuberculosis (TB) is the leading cause of death in patients infected with HIV in economically poor settings.^1^ HIV infection is the strongest risk factor for TB and over four million people are coinfected with both organisms, the majority of whom reside in Africa.^2^ Such coinfection worsens the prognosis of HIV infection by increasing viral replication^3^, ^4^, ^5^ and may result in subsequent immune suppression^6^ and a higher risk of acquiring other, potentially lethal, opportunistic infections.^7^

To mitigate this risk, WHO recommended countries to introduce isoniazid preventive therapy (IPT), INH 300 mg daily for 6 months, for people living with HIV^8^ aimed at preventing and reducing incidences of TB. Isoniazid (INH) has been in use since many years as prophylaxis of TB and has been associated with small risk of fatal hepatotoxicity that ranges from 0.003% to 0.06%.^9^, ^10^, ^11^ Liver injuries with fatal outcomes have been reported to the US‐CDC^12^ and other published case reports^13^, ^14^ also reported fatal hepatotoxicity associated with INH preventive therapy. Age >35 years,^15^, ^16^ alcohol consumption^11^, ^17^ and concurrent administration of other hepatotoxic agents^18^ are identified as possible risk factors for the INH‐induced hepatotoxicity. Following the introduction of IPT in patients taking highly active anti‐retroviral therapy (HAART), some studies reported little risk of hepatotoxicity ranging from 0.07% to 1.9% without fatal outcomes.^19^, ^20^, ^21^, ^22^ Considering the low risks of INH‐induced hepatotoxicity, neither the WHO nor the Eritrean national IPT guidelines recommend routine baseline liver function test (LFT) for IPT.^23^, ^24^

However, when the policy of IPT for PLWH was introduced and scaled up in Eritrea in 2014, frequent IPT‐associated hepatotoxicity and fatality have been reported to the Eritrean Pharmacovigilance Centre. Many of the case reports showed that patients were stable under HAART for several years and their liver enzymes were normal prior to initiation of IPT. Therefore, the aim of the study is to assess the causal association of IPT and hepatotoxicity, identify possible risk factors and assess the preventability in patients on HAART.

## 2 MATERIALS AND METHODS

This is a retrospective descriptive study of cases spontaneously reported to the Eritrean Pharmacovigilance Centre. All hepatotoxicity cases associated with IPT, reported between June 2014 (introduction of IPT) and June 2016 were retrieved from the Eritrean adverse drug reaction database and exported to excel spread sheet for descriptive and qualitative analysis. For more detail, individual case reports (yellow forms) were also retrieved based on their unique ID from the archives of the Eritrean Pharmacovigilance Centre. Cases were then summarized in an excel spreadsheet according to their age, sex, body weight, daily dose taken, type of HAART given, other concomitant/cosuspected drugs used, time to reaction onset, causality, preventability, baseline data (LFT as well as existence of hepatitis B and C), seriousness, severity, management or treatment taken, and reaction outcome for analysis.

Causality was reassessed using Naranjo probability scale^25^ and then subjected to Austin Bradford‐Hill criteria^26^ for further case series assessment. The P‐Method developed by the Moroccan Pharmacovigilance Centre in collaboration with the WHO was also used to assess preventability of the individual case safety reports.^27^ The strength of the association was measured using proportional reporting ratio and seriousness of the reaction was determined according to the definition of the ICH E2A guideline^28^ and severity of liver toxicity was assessed based on the US‐FDA drug‐induced liver toxicity grading scale^29^ as follows:

- Mild hepatotoxicity is defined as AST or ALT elevations of 1.1‐2.5 times the upper limit normal (ULN) (<121 IU/L).
- Moderate hepatotoxicity is defined as elevation of AST/ALT of 2.6‐5.0 times the ULN (121‐200 IU/L).
- Severe hepatotoxicity is the elevation of AST/ALT of 5.1‐10 times of the ULN.
- Very severe (potentially life threatening) can be defined as the elevation of AST/ALT >10 times ULN.

## 3 RESULTS

In the period of June 2014 to June 2016, a total of 60 hepatotoxicity cases were spontaneously reported to the Eritrean Pharmacovigilance Centre with 31 of them related to IPT in patients on HAART. The cases were eight males and 23 females with a median age of 43 years (ranging from 25 to 67 years) (Table 1) and median body weight of 46 kg. Liver Function Test (LFT) baseline during the initiation of IPT was normal in 19 cases and unknown in 12 cases. Hepatitis B and C coinfections were ruled out in 24 of the cases and results were unknown in seven cases (Table 1).

**Table 1:** Distribution of the cases according to their background characteristics

Background characteristics	N(%)
Age at index date	
<45 years	19 (61.3)
45‐<60 years	9 (29.0)
>=60 years	3 (9.7)
Sex	
Male	8 (25.8)
Female	23 (74.2)
Body weight at IPT initiation (all adults)	
<=40 kg	7 (22.6)
40‐50 kg	12 (38.7)
>50 kg	9 (29.0)
Unknown	3 (9.7)
Status of hepatitis B and C coinfection at IPT initiation	
Positive	0 (0.0)
Negative	24 (77.4)
Unknown	7 (22.6)
LFT baseline at IPT initiation	
Normal	19 (61.3)
Deranged	0 (0.0)
Unknown	12 (38.7)
Type of HAART	
Nevirapine‐based regimen	23 (74.2)
Efavirenz‐based regimen	8 (25.8)

IPT, isoniazid preventive therapy; LFT, liver function test; HAART, highly active anti‐retroviral therapy.

Out of the total cases, 23 received Nevirapine containing and the rest eight took Efavirenz containing HAART regimen. Patients took vitamin B6 concurrently with IPT and HAART in all the cases with no other concomitant medicines. The time to reaction onset was reported in 22 cases with a median time to onset of 61 days (ranging from 14 to 304 days) (Table 2). All but six (80.6%) of the cases were marked as “serious.” Reason for seriousness was reported as life threatening (n = 15), hospitalization (n = 6), and death (n = 4). Majority of the hepatotoxicity cases were either severe or very severe (Table 3). In 83.9% of the cases, reaction was reported as either “recovered” or “recovering” (Table 2). In the cases with fatal outcomes, three were taking Nevirapine containing HAART and one was taking Efavirenz‐based regimen.

**Table 2:** Summary of hepatotoxicity cases associated with IPT in HIV patients on HAART

S. No	Sex	Age	Other Suspected (S) or Concomitants (C)	Reported reaction	Time to onset (days)	Severity	Reaction outcome
1	F	30	Vitamin B‐6 (C)	Hepatotoxicity	60	Severe	Recovered
2	F	46	Vitamin B‐6 (C)	Hepatotoxicity	N/A	Very severe	Recovered
3	F	35	Vitamin B‐6 (C)	Hepatotoxicity	N/A	Severe	Recovered
4	F	42	Vitamin B‐6 (C)	Hepatotoxicity	N/A	Very severe	Recovered
5	M	67	Vitamin B‐6 (C)	Hepatotoxicity	N/A	Moderate	Recovered
6	M	41	Vitamin B‐6 (C)	Hepatotoxicity	N/A	Very severe	Fatal
7	F	37	Vitamin B‐6 (C)	Hepatotoxicity	N/A	Severe	Recovered
8	F	34	Vitamin B‐6 (C)	Hepatotoxicity	77	Very severe	Recovering
9	F	44	Vitamin B‐6 (C)	Hepatotoxicity	55	Very severe	Recovering
10	M	49	Vitamin B‐6 (C)	Hepatotoxicity	61	Severe	Recovering
11	M	42	Vitamin B‐6 (C)	Hepatotoxicity	76	Severe	Recovering
12	M	46	Vitamin B‐6 (C)	Hepatotoxicity	14	Moderate	Recovered
13	F	44	Vitamin B‐6 (C)	Hepatotoxicity	93	Very severe	Recovered
14	F	25	Vitamin B‐6 (C)	Hepatotoxicity	14	Unknown	Recovered
15	F	61	Vitamin B‐6 (C)	Hepatotoxicity	14	Unknown	Recovered
16	F	49	Vitamin B‐6 (C)	Hepatotoxicity	60	Unknown	Recovering
17	F	44	Vitamin B‐6 (C)	Hepatotoxicity	34	Unknown	Fatal
18	M	66	Vitamin B‐6 (C)	Hepatotoxicity	90	Unknown	Fatal
19	M	42	Vitamin B‐6 (C)	Hepatotoxicity	73	Unknown	Recovered
20	M	47	Vitamin B‐6 (C)	Hepatotoxicity	60	Very severe	Not yet recovered
21	F	49	Vitamin B‐6 (C)	Hepatotoxicity	43	Very severe	Recovering
22	F	35	Vitamin B‐6 (C)	Hepatotoxicity	304	Severe	Recovering
23	F	36	Vitamin B‐6 (C)	Hepatotoxicity	101	Very severe	Recovering
24	F	43	Vitamin B‐6 (C)	Hepatotoxicity	120	Severe	Recovering
25	F	41	Vitamin B‐6 (C)	Jaundice	19	Unknown	Recovered
26	F	39	Vitamin B‐6 (C)	Hepatotoxicity	83	Very severe	Recovered
27	F	34	Vitamin B‐6 (C)	Hepatotoxicity	83	Very severe	Recovering
28	F	37	Vitamin B‐6 (C)	Hepatotoxicity	N/A	Mild	Recovered
29	F	51	Vitamin B‐6 (C)	Hepatotoxicity	N/A	Very severe	Recovered
30	F	45	Vitamin B‐6 (C)	Hepatotoxicity	N/A	Unknown	Fatal
31	F	56	Vitamin B‐6 (C)	Hepatotoxicity	38	Unknown	Recovering

**Table 3:** Severity of hepatotoxicity cases

S. No.	Severity	Count
1	Mild	1
2	Moderate	2
3	Severe	7
4	Very severe	12
5	Unknown	9
Total		31

Dechallenge information was reported in 22 of the cases and reactions abated in all the cases following withdrawal of INH. INH was subsequently reintroduced in one case and reaction reappeared with rechallenge. In the rest nine cases, dechallenge and rechallenge information was unknown. In majority of the cases (71%), the causal association between INH and hepatotoxicity was found to be “probable” and in the rest “possible.”

To manage the hepatotoxicity, INH was stopped in 83.9% of the cases (Table 4). Moreover, in 15 of the cases HAART was either switched (n = 12) or temporarily stopped (n = 3). In 61% of the cases, hepatotoxicity was preventable because of an apparent overdose of INH on a weight‐based calculation. For the rest 12 cases, nine were nonpreventable and three were nonassessable.

**Table 4:** Management of the adverse drug reactions

S. No.	Action taken	Count
1.	Only INH stopped	13
2.	INH stopped and HAART switched	10
3.	INH and HAART stopped	3
4.	HAART switched	2
5.	No action taken	3

The results of causality assessment with the Austin Bradford‐Hill Criteria are explained in detail on Table 5.

**Table 5:** Causality assessment using the Austin Bradford‐Hill criteria

Criterion	Outcome
Strength of association	Proportional reporting ration (PRR) is 14.6; thus indicating a strong statistical signal
Consistency of cases	Reactions reported from different health facilities were similar in their clinical features. It was confirmed that reactions manifested following introduction of IPT in all the cases. Majority of the reports showed that patients were stable when under HAART for several years and their liver enzymes were normal prior to initiation of IPT
Specificity of the association	Hepatotoxicity was the only reported adverse reaction except in one case (ie, anemia coreported). In all the cases, INH was the only suspected drug; HAART as well as B6 were the only concomitant drugs reported (Table 2)
Temporal relationship	All reactions manifested after INH was administered and the median time to reaction onset was 61 days
Dose‐response relationship	About 68% of the cases were taking 300 mg though their body weight was less than or equal to 50 kg. In 22 of the cases, reactions abated following withdrawal of INH
Plausibility of the association (plausible mechanism)	Through acetylation by N‐acetyltransferase, hydrolysis and the Cytochrome P450 enzymes, INH can be metabolized in the liver producing acetylhadrazine and hydrazine. These metabolites are capable of participating in reactions that generate oxidative stress which subsequently cause hepatotoxicity.^30^ Furthermore, Isoniazid has an inhibiting effect on CYP1A2 activity, which is suggested to be involved in hydrazine detoxification. Hence, Isoniazid can induce its own toxicity, possibly by the induction or inhibition of this enzyme.^31^, ^32^
Experimental evidence	Before initiation of IPT, LFT baseline was normal in 61.3% and hepatitis B and C coinfections were ruled out in 77.4% of the cases. Positive dechallenge was reported in 71.0% of the cases with one positive rechallenge
Coherence	INH is well known to cause Hepatotoxicity
Analogy	N/A

## 4 DISCUSSION

Our study on causality assessment using Austin Bradford‐Hill criteria showed that the cases were consistent and the association was specific. Although the cases were reported from different healthcare professionals and health facilities, their clinical features were more or less similar, mainly manifested in patients greater than 35 years old. INH was the only suspected drug and HAART as well as vitamin B6 were the only concomitant drugs reported in all cases. Besides, in all but one, hepatotoxicity was the only reported reaction which shows specificity of the cases. The association seems strong as the proportional reporting ratio (PRR) was found to be 14.6. This tells us that hepatotoxicity was reported more than 14 times (among all reactions reported) for INH compared to other drugs in the Eritrean Pharmacovigilance database. PRR greater than two indicates that there is a statistical signal which, however, does not necessarily mean a true safety signal.

The normal LFT baseline results prior to the initiation of INH in majority of the cases shows that patients were stable with HAART for several years and patients developed hepatotoxicity shortly following administration of IPT. This provides a plausible temporal relationship between IPT and hepatotoxicity. It also provides an impression that the hepatotoxic effect was least likely to be attributed to HAART. Furthermore, the median time to onset of the hepatotoxic effect from the initiation of IPT was consistent with findings from previous studies.^15^, ^33^ Majority of our cases seem to be overdosed since their weight was less than or equal to 50 kg and they were taking 300 mg of INH daily. Following withdrawal of INH, reaction abated within few weeks in 22 of the cases and recurred in one case after reintroduction of INH. This provides a plausible dose‐response relationship and strengthens the causal association. Alternative causes like hepatitis B and C and other hepatotoxic drugs intake were ruled out in majority of the cases.

The high PRR, the plausible temporal relationship, the consistency of the cases and specificity of the association, the positive dechallenge in several cases and rechallenge in one case, the positive dose‐response relationship, the plausible biological mechanism,^30^ and the unavailability of other reported alternative explanations of the hepatotoxic effect are therefore evidences that support a causal association between IPT and hepatotoxicity. This study, however, had many limitations. Reactions were spontaneously reported from healthcare professionals thus true denominator data cannot be determined. Taking the inherent underreporting of ADRs into account, the cases do not reflect the exact incidence of INH‐induced hepatotoxicity in Eritrea. Besides, baseline liver function test, hepatitis B and C as well as alcohol consumption status were not ruled out in some patients.

Since Eritrea started to implement IPT in 2014 until June 2016, there were a total of 4919 patients enrolled into IPT.^34^ Of these, 4398 patients have already completed the 6‐month duration therapy and 188 have interrupted treatment due to adverse drug reactions. These voluntarily reported hepatotoxicity cases were therefore among the ADRs that caused treatment interruptions but do not reflect the exact incidence rate in the country due to under reporting. Several of the hepatotoxicity cases were found to be severe or very severe including four cases with fatal outcomes. This is not consistent with previous studies conducted on safety of INH as none of the previous publications reported fatal outcomes.^19^, ^20^, ^21^, ^22^

It is already known that INH‐induced hepatotoxicity increases with increased age (particularly in individuals greater than 35 years old) compared to younger adults.^15^, ^16^, ^35^ Our preliminary finding also supports the previous knowledge as above 80% of our cases were older than 35 years. To mitigate such problems, the American Thoracic Society recommends^35^ “baseline and follow‐up ALT tests for patients >35 years old either on monthly, bi‐monthly or at 1, 3, and 6 months, depending on perceived risk and ALT stability.” In our case, had there been close LFT monitoring, majority of the complicated cases including those with fatal outcomes could have been prevented.

Due to the rarity of INH‐induced hepatotoxicity, routine laboratory monitoring is not recommended by the WHO.^23^ It should, however, be noted that a deferred diagnosis of a fivefold and greater transaminases elevation or a threefold and greater elevation with symptoms is associated with high mortality risk (mainly due to hepatic failure) that may exceed 50%, unless liver transplantation is performed.^36^ Signs and symptoms of hepatotoxicity, liver failure, and eventually hepatic encephalopathy usually present late, making it harder for earlier detection of organ damage, especially if routine laboratory monitoring is not in place.^37^, ^38^, ^39^

Our preliminary assessment therefore concluded that HIV patients taking IPT are at risk of INH‐induced hepatotoxicity that may end up with fatal outcomes some of which could possibly be preventable. The INH‐induced hepatotoxicity caused treatment interruptions even in previously stable patients with HAART for several years. Older age greater than 35 years, inappropriate laboratory monitoring and over dose were identified as possible risk factors of INH‐induced hepatotoxicity. Continuous education to patients and healthcare workers about the risk of INH‐induced hepatotoxicity and being vigilant during clinical visits is highly recommended to identify the problem at the earliest possible time. To manage the adverse effect, clinicians must stop INH, observe patients closely and provide symptomatic treatment if required.^40^ They should also have a high level of suspicion that herbal and dietary supplements are implicated in injury. Taking the limitations of clinical monitoring stated above into account, the need for routine laboratory monitoring and effectiveness/risk assessment should be considered with highest standards of epidemiologic studies in clinical practice.

## ACKNOWLEDGEMENTS

The authors would like to acknowledge the following reporters for their vigilance and commitment: Aman Solomon, Berhane Gbereyohannes, Danait Andehaimanot, Dina Medhanie, Hirity Kiflay, Luul Tesfamichael, Teklezghi Teklesenbet, Theodros Tekeste, Yafiet Hailemichael, and Yordanos Ghebrekidan.

## DISCLOSURES

The authors declare that they have no competing interests and no source of funding was used to carry out the study.

## AUTHORS CONTRIBUTIONS

All of the authors played a key role in the analysis and interpretation of the cases. MR, MD, and MZ drafted the article and it was edited by the rest of the authors. All authors give their consent for publication.
